# Meta-analysis of the effects of dance- and movement-based kinesthetic games on cognitive function in older adults with cognitive impairment (CI) under different intervention periods

**DOI:** 10.1007/s40520-025-03233-y

**Published:** 2025-12-11

**Authors:** RuYan Zhang, BingHong Gao, Ran Li, RuiXia Zhou

**Affiliations:** 1 Macao Polytechnic University, Macao, China; 2 Shanghai University of Sport, Shanghai, China; 3 Shandong Sport University, Shandong, China

**Keywords:** Exergames, Older adults, Cognitive impairment, Meta-analysis

## Abstract

**Objective:**

To evaluate the effects of different types and durations of exergame interventions on cognition in elderly individuals with cognitive impairment (CI) through a meta-analysis.

**Methods:**

A computerized search was conducted in databases including Cochrane Library, PubMed, Web of Science, Embase, and CNKI from database inception to September 2025. Quality assessment and data extraction were performed on the included studies. Results measures included the MoCA , MMSE, etc. The study designs were randomized controlled trials or qua-si-experimental studies. The Cochrane Handbook’s risk of bias tool was used for quality assessment, and Rev Man 5.0 software was employed to conduct meta-analyses on the ef-fects of dance-based and movement-based exergames on cognitive function in elderly CI patients.

**Results:**

A total of 13 studies involving 433 CI patients were included. Analysis of the 13 studies showed that dance-based exergaming was significantly superior to exercise-based exergaming in cognitive ability (SMD = 0.73, P < 0.01); interventions lasting ≥8 weeks were more effective than those lasting <8 weeks (SMD = 1.01, P = 0.02).

**Conclusion:**

Dance-based exergames significantly improve cognition in elderly individu-als with CI, with better effects observed in long-term interventions. However, more high-quality studies are needed to verify these findings and to supplement analyses on ex-ercise dose effects.

**Supplementary Information:**

The online version contains supplementary material available at 10.1007/s40520-025-03233-y.

## Introduction

With the rapid development of population aging in China, the number of dementia patients has multiplied, becoming a serious health and social issue and a major challenge faced by an aging society [1]. However, there is currently a lack of effective treatment methods; drug efficacy is not significant, and safety remains to be verified, so non-pharmacological interventions have become a popular research topic in recent years [1]. Studies have shown that neuroplasticity and cognitive compensation ability may be the basis for the effectiveness of non-pharmacological interventions [1]. Therefore,somatosensory games, which provide multisensory stimulation through virtual reality technology combined with physical activity and cognitive challenges, may become an effective non-pharmacological intervention method. Research shows that somatosensory games can improve executive function, working memory, and balance ability in the older adults. However, there is still a lack of systematic comparisons of the effects of different types of games and different intervention durations on patients with varying degrees of cognitive impairment [2, 3].

At the same time, past meta-analytic studies mostly treated exergames as a whole and did not fully account for differences between different types of exergames. In terms of type selection, dance and action exergames have unique advantages for improving specific cognitive domains. Meta-analyses show that dance games significantly improve executive function and episodic memory (effect size d = 0.68), which is directly related to their complex multitasking demands [4] action games are more prominent in improving processing speed and visuospatial abilities (effect size d = 0.49) [5]. In contrast, while competitive games can also improve certain cognitive functions, their effects are often accompanied by increased anxiety levels (BAI score increased by 2.3 points), which is not conducive to long-term cognitive health [6]. FNIRS monitoring shows that action games increase prefrontal oxygenated hemoglobin concentration by 12.3μM, significantly above baseline levels [7]. EEG studies have found that increases in θ-band power in the dance game group are positively correlated with memory improvement (r = 0.62) [8]; these neuroplasticity indicators were not significantly changed in interventions using competitive games. At the same time, some researchers have pointed out that competitive exergames pose obvious physiological stress risks for older adults. These games typically include sudden stimuli and rapid response demands, which may trigger sharp heart rate increases (average increase 35 bpm); such stress not only offsets cognitive benefits but may also induce negative emotional states such as anxiety [3], exceeding the safety range for older adults [9]. Regarding the choice of intervention duration, 8 weeks is a key dividing line for exercise interventions, with solid theoretical and practical foundations. Short-term interventions under 8 weeks are suitable for quickly achieving specific functional improvements, assessing individual responsiveness, or serving as the initiation phase of a long-term intervention [10–15]. These interventions typically use higher frequency and intensity, focusing on neuromuscular adaptation, skill learning, and immediate psychological benefits [16–20]. In contrast, medium- to long-term interventions over 8 weeks can produce more comprehensive physiological adaptations, metabolic improvements, and stable behavioral changes [21–25].

Therefore, this paper selected the two types of exergames that showed significant intervention effects and are beneficial to cognitive health (dance and action) for meta-analysis, to explore in depth the uniqueness of these two types of exergames and their differential effects on cognitive function in older adults with different types of cognitive impairment. For example, dance exergames may have advantages in improving mood, social function, and music-related cognitive abilities (such as sense of rhythm and melody memory), while action exergames may be more effective in improving reaction speed, spatial cognitive abilities, and executive function[26–30]. At the same time, the choice of intervention duration should be individualized based on the characteristics of the target population, expected outcomes, and available resources [31–35]; different cognitive levels will also produce different effects in the choice of intervention duration. Therefore, such a refined analysis helps select more appropriate exergame intervention types and durations for older adults with different types and severities of cognitive impairment [3, 31], achieving the goal of personalized treatment and maximizing improvements in cognitive function for older adults with cognitive impairment [36–41]. To this end, this paper takes dance and action exergames and different intervention durations—game types and durations that are closely related to cognitive function and whose research results are controversial—as the main research content, to provide some theoretical reference for research aimed at enhancing the cognitive abilities of older adults with cognitive impairment[42–46].

## Subjects and methods

### Search strategy

Chinese databases: CNKI [47]; Foreign databases: Cochrane Library, PubMed, Web of Science, Embase, etc. The search used the following MeSH terms: (((Exercise intervention methods (“virtual reality”[Mesh] OR “video games”[Mesh] OR “VR” OR “active video game” OR “interactive video game” OR “motion sensing game” OR “video game” OR “exergaming”[Mesh] OR “Eye Toy” OR “somatosensory game” OR “Wii sports” OR “Kinect Sports”)) AND (Intervention population (“older”[Mesh] OR “older adults” OR “elderly” OR “Aged”[Mesh])) AND (Characteristics of the population (“dementia” [Mesh] OR “Alzheimer” OR “Alzheimer syndrome” OR “Alzheimer disease” [Mesh] OR“Cognitive impairment”[Mesh] OR“Mild cognitive impairment”OR“MCI” [Mesh] OR“Parkinson Disease”[Mesh])) AND (Outcome indicators (“cognitive ability” OR “cognitive impairment”[Mesh] OR “Memory”[Mesh] OR “Attention”[Mesh] OR “Execution ability” OR “Reaction time”[Mesh]))) among other keywords, combined in various ways across databases to find literature. Additionally, relevant systematic reviews or meta-analyses were checked for included studies and traced [48]. The search period was from database inception to Sep 2025.

### Inclusion and exclusion

Inclusion criteria: (1) Clinically diagnosed cognitive impairment, mild cognitive impairment (MMSE score 21–26) [49]or dementia in elderly people aged 60 years and older [50]. (2) Experimental group intervention: interactive exercise–cognitive training; (3) Control interventions: may include single-task physical or cognitive activities, usual care, waitlist, health education, sham training [50]; (4) Outcome measures include [Mini-Mental State Examination (MMSE) score, Alzheimer’s Disease Assessment Scale–Cognitive Subscale (ADAS-COG) score, Montreal Cognitive Assessment (MoCA-JL), Frontal Assessment Battery (FAB)], activities of daily living [Activities of Daily Living (ADL) score, Kenzie’s Self-Care Evaluation (KSCE) score], TMT-A (Trail Making Test A), TMT-B (Trail Making Test B), Geriatric Depression Scale (GDS) score, Cornell Scale for Depression in Dementia (CSDD) score, executive function [10]; (5) quasi-experimental study design; (6) Chinese and English literature; (7) duplicate reports were combined using the snowball method [51].

Exclusion criteria: (1) Study subjects are healthy elderly or non-CI patients (e.g., isolated anxiety/depression), or diagnosis unclear; (2) Experimental group intervention is not a motion-sensing game or operational parameters are not specified, or the control group received cognitive-related interventions (e.g., drugs, transcranial magnetic stimulation); (3) Cognitive-related outcome measures were not reported or non standardized instruments were used; (4) Noninterventional studies (e.g., observational studies, reviews), duplicate publications, or articles with unavailable data; (5) Intervention duration < 4 weeks [52, 53].

### Literature screening and quality assessment

After de-duplication with Endnote, two researchers independently and blindly extracted and screened the relevant indicators, with disputes resolved by a third party. Extracted items included: first author of the study, year of publication, cognitive impairment, sex, age, sample size, type of exergame, experimental design scheme, etc. For studies lacking data or with unclear information, authors were contacted by email to obtain details [54]. Quality assessment was performed using the Cochrane risk of bias tool (RevMan5.4). The Cochrane risk of bias assessment tool was used to evaluate the included studies, covering seven items: generation of random sequences, allocation concealment, blinding, description of withdrawals, completeness of outcome data, selective outcome reporting, and other biases. The included studies were evaluated on these seven items: generation of random sequences, allocation concealment, blinding, description of withdrawals, completeness of outcome data, selective outcome reporting, and other biases [54]. If all seven items met criteria they were rated A, indicating low risk of bias; some items meeting criteria were rated B, indicating moderate risk of bias; if none of the seven items met criteria they were rated C, indicating high risk of bias. Overall quality was graded A/B/C based on the number of low, moderate, high, and unclear risks [53].

### Statistical methods

Using Rev Man 5.4 software, continuous data were expressed as mean difference (MD) or standardized mean difference (SMD) with 95% CI. Heterogeneity was assessed using the P value and I^2^ (a fixed-effect model was used when P >0.10 and I^2^ < 50%; otherwise a random-effects model was used). When heterogeneity was significant, subgroup analyses, sensitivity analyses, or descriptive analyses were conducted. The significance threshold was set at *P < 0.05*.

### Grade results

Use GRADE pro 3.2 to rate the quality of evidence for the results of the meta-analysis. The rating criteria include risk of bias, imprecision, inconsistency, indirectness, and other factors. RCTs start as high quality; failing any criterion downgrades by one level. ●●●● indicates high-quality evidence, while ●○○○ indicates very low-quality evidence [55].

## Results

### Literature search results

**Fig. 1:**
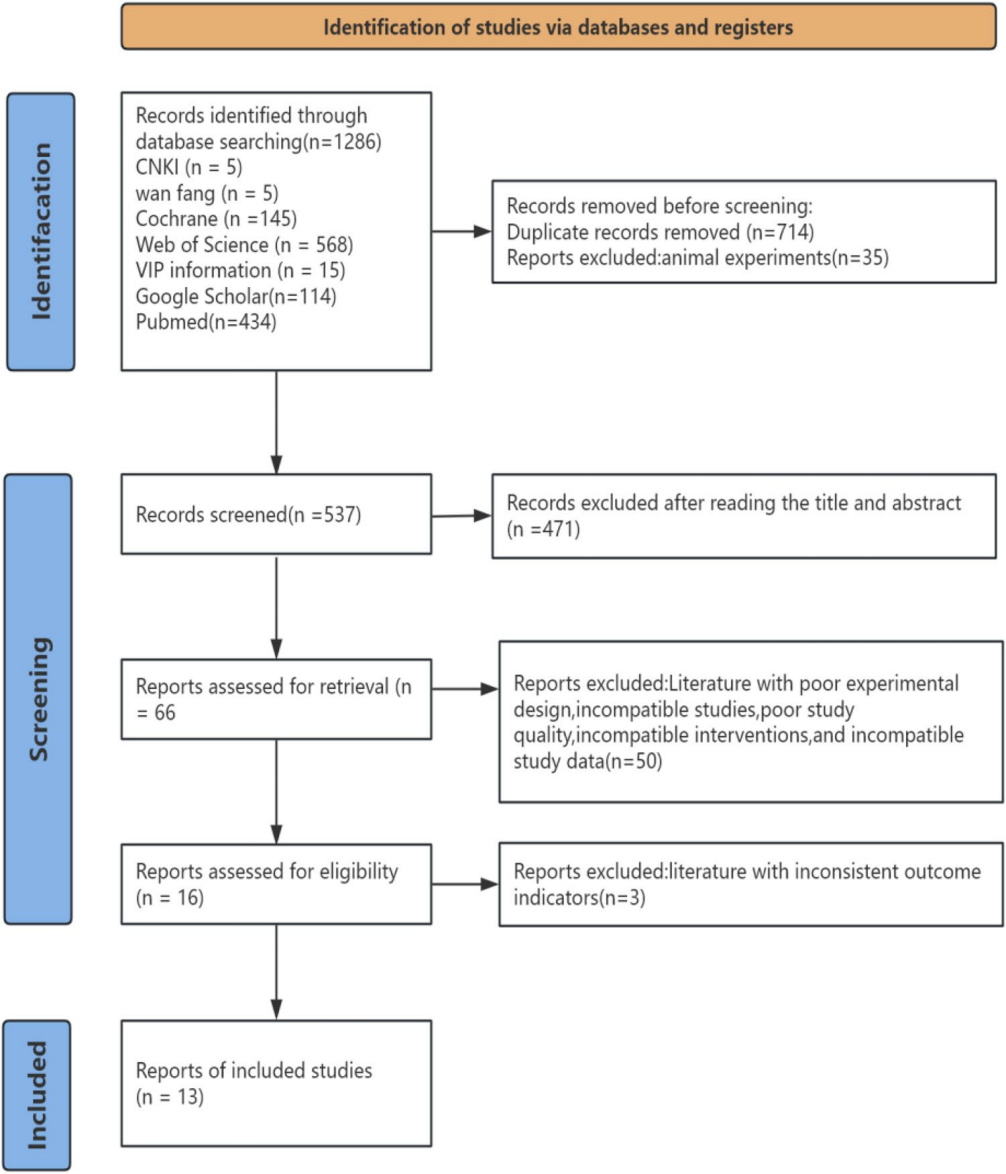
Flowchart of study selection

This study collected relevant literature through searches, including 1276 foreign language articles and 10 Chinese articles, totaling 1286 articles. Using the literature management tool EndNote, 714 duplicate articles were removed, as well as meta-analyses and systematic reviews. Along with35articles on animal experiments, there were537remaining articles. Subsequently, by reading the titles and abstracts, 471 articles unrelated to the research topic were further excluded, preliminarily selecting 66 potentially relevant articles. Excluding 50 articles due to poor experimental design, inconsistent research content, low research quality, incompatible intervention methods, or inconsistent research data. After further in-depth reading of the full texts, based on pre-established inclusion and exclusion criteria, 3articles with inconsistent outcome indicators were excluded, finally including 13 articles involving a total of 577experimental subjects. The literature screening flowchart is shown in Fig 1, and the basic characteristics of the included articles are shown in Table 1.

**Table 1:** Basic Characteristics of Included Literature

First author	Region	Published (year)	Disease	Group (Sample Size)	Intervention measures	Treatment plan	Outcome in dictators
Giuseppe Pichierr [9, 30]	Switzerland	2012	MCI	Treatment(11) Control (11)	Strength and balance training + dance video game training Only traditional strength and balance training are accepted	2 times/week, for a total of 12 weeks	FPA , Gait Performance Gaze Behavior, Dual Task Costs, MoCa、TMT
Jahouh et al 2021 [15, 16]	Brazil	2019	AD	Treatment(8) Control (8)	Training using the Xbox 360 Kinect TM game device	2 times/week, for a total of 5 weeks, each training session lasts 45 to 60 minutes	MoCA, MMSE, FAB,MDS- UPD,PDQ-39
Katsunari Sato [3, 21]	Japan	2023	MCI	Treatment(10) Control (11)	Dance video game training using Step Mania 3.9	1 time/week, 5 times/week, for a total of 12 weeks	MMSE, MoCA、TMT, Stroop
Mosayeb Mozafari, PhD [4, 26]	Iran	2025	AD	Treatment (30) Control (30)	Motion-sensing games: golf and archery	3 times/week, 45 minutes each time, for a total of 12 weeks	MMSE、PSA、MoCa
Nadia Rizki Rahmawati [5, 27]	Indonesia	2024	MCI	Treatment (12) Control (14)	Boxing workout game using Xbox 360® Kinect (5 minutes warm-up, about 15 minutes boxing workout game, remaining time for cool down) + low-intensity aerobic exercise 5 days a week, 15 minutes each time (5 minutes warm-up + core exercise + cool down phase) No boxing workout game, only low-intensity aerobic exercise (5 minutes warm-up + core exercise + cool down phase)	3 times/week, for 8 weeks, 25 minutes each time	MoCA
Pauline Maillot [6, 28]	France	2012	AD、MCI	Treatment(15) Control(15)	Warm-up + motion-sensing game training using Nintendo Wii console + relaxation group	2 times/week, 1 hour each time, for 12 weeks, total 24 hours	Trail Making Test, Stroop Color Word Interference Test, Letter Sets Test, Matrix Reasoning , Test of Directional Headings Test
Lim et al 2023 [3, 7, 29]	China Taiwan	2020	AD	Treatment(12) Control(12)	The first 6 weeks involve IVGB (Interactive Video Game-Based) exercise training, followed by no IVGB exercise in the next 6 weeks. Exercise intervention for the first 6 weeks (control phase), followed by IVGB training in the next 6 weeks (intervention phase)	3 times/week	MMSE, MoCa, BBS、MDRT, MSL, SF-36
Schoene, Daniel [12, 33]	United States	2014	MCI	Treatment(15) Control(17)	Using an interactive training system combining electronic pedals and a computer interface	1.5 h per session, 1 session per week, a total of 20 weeks	MMSE, MoCa ,RBMT, TEA, ROCFT
suwicha Jirayucharoensa [8, 17]	Thailand	2019	MCI	Treatment(36) Control(20)	Conventional exercise and care Conventional care + exercise-based game (Microsoft Kinect v2) training group, Conventional exercise and care	2-3 times per week, 30 minutes each time, a total of 20 sessions	SWM, CANTAB
Zheng Jiaying [12]	China	2018	MCI	Treatment(18) Control(20)	Microsoft Kinect 2.0 motion interactive device, XBOX 360 Fruit Ninja game Conventional exercise and care	5 times per week, 1 hour each time, for 8 weeks, with 10 minutes of sensory games each session	MMSE, CSDD, MoCa
Nathalie Swinne [13, 14]	United Kingdom	2021	CI	Treatment(23) Control(22)	“Dividat Senso” Watch your favorite music videos	3 times per week for 8 weeks, 15 minutes each time	SPPB, SRTT, MoCA, NPI, MMSE, CSDD, ADL
Maha Jahouh [56]	Spain	2021	CI	Treatment(23) Control(22)	Use the Nintendo Wii Fit console for rehabilitation training	3 times a week ,for 8 weeks then 2 times a week, alternating for a total of 20 rehabilitation sessions	MCE、FAST-GDS、DAIR、EGD-15、EADG
Hafsah Arshad [15, 49]	Pakistan	2021	MCI	Treatment (18) Control (0)	Kinect-based virtual reality game “Body and Brain Exercises”	6 weeks of cognitive training, 30 minutes each session, including 5 minutes of warm-up and 5 minutes of relaxation time	MMSE, MoCA, TMTA, B ,Language Fluency Test

FPA = Foot Placement Accuracy Test, Gait Analysis = Gait Analysis, Fear of Falling = Fear of Falling Assessment, Gaze Behavior = Gaze Behavior, MoCA = Montreal Cognitive Assessment, FAB = Frontal Assessment Battery, MDS-UPDRS = Movement Disorder Society Unified Parkinson’s Disease Rating Scale, PDQ-39 = Parkinson’s Disease Questionnaire, MMSE = Mini-Mental State Examination, TMT = Trail Making Test, Stroop Test = Stroop Test, FNIRS = Functional Near-Infrared Spectroscopy, PSQ = Problem Solving Questionnaire, GDS-SF = Geriatric Depression Scale Short Form, PANAS = Positive and Negative Affect Schedule, SUS = System Usability Scale, PASE = Physical Activity Scale for the Older adults, Perceived Stress Scale = Perceived Stress Scale, GDS = Geriatric Depression Scale, Functional Fitness Test = Functional Fitness Test, Executive Control Tasks = Executive Control Tasks, Visuospatial Tasks = Visuospatial Tasks, Processing Speed Tasks = Processing Speed Tasks, BBS = Berg Balance Scale, MFES = Modified Falls Efficacy Scale, MDRT = Multidirectional Reach Test, MSL = Maximum Step Length Test, SF-36 = 36-Item Short Form Health Survey, Hand Reaction Time = Simple Reaction Time Test, Digit-Letter Test = Digit-Letter Test, CSRT = Choice Stepping Reaction Time Test, ANT = Attention Network Test, Icon-FES = Icon Falls Efficacy Scale, Mini-Cog = Mini-Cognitive Test, SWM = Spatial Working Memory, DMS = Delayed Match to Sample, RVP = Rapid Visual Information Processing, TMSE = Thai Mental State Examination, MCE= Lobo Mini Cognitive Check, DAIR= Apathy Interview and Rating for Dementia, EGD-15= Geriatric Depression Scale, EADG= Depression scale

### Quality assessment

**Fig. 2:**
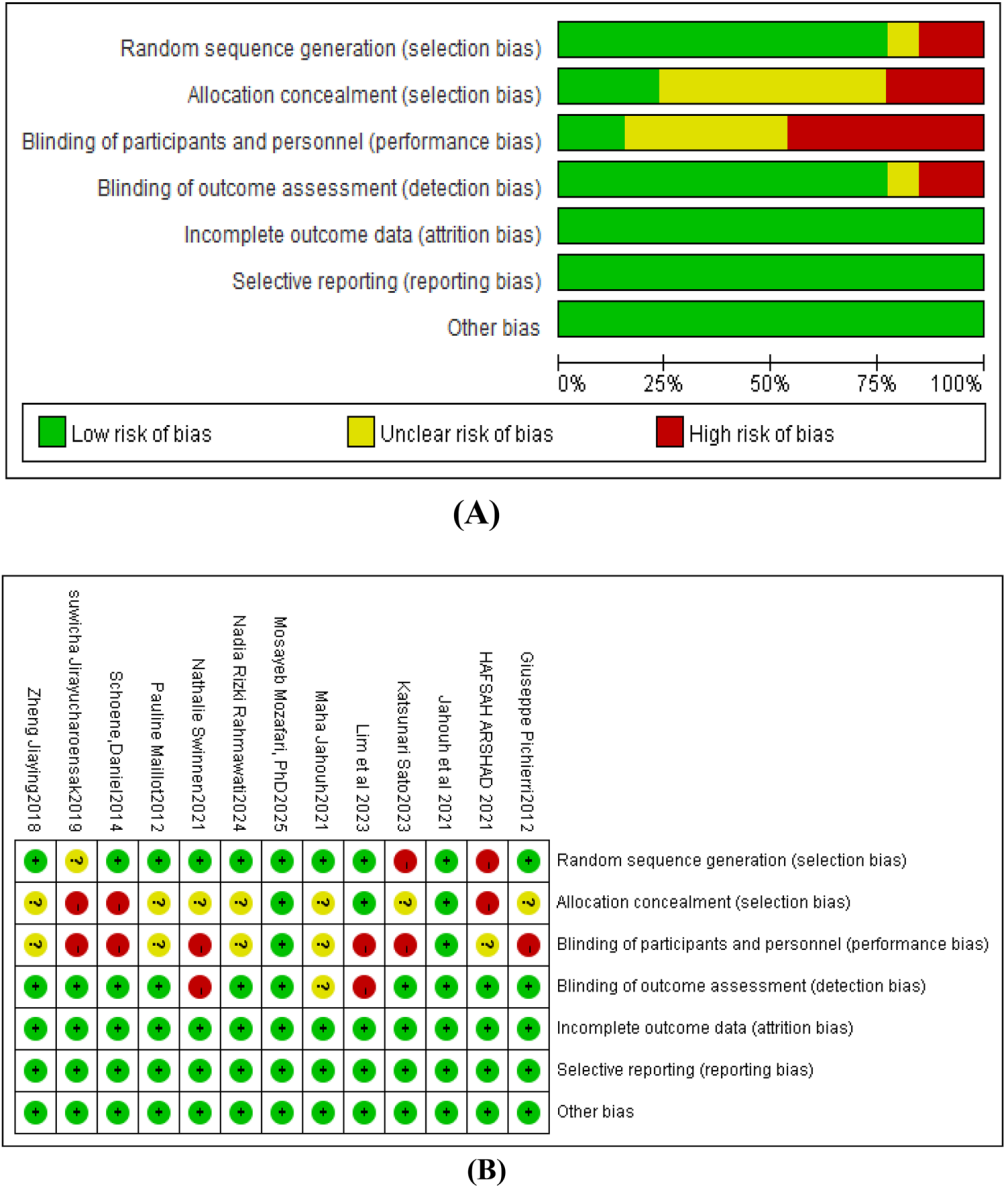
Traffic light plot for assessment of risk of bias of each included random ized trial (**A**) and weighted plot for assessment of overall risk of bias B using CochraneRoB2tool [3–16, 56]] (**B**) (n=13studies). Traffic light plot reports five risk of bias domains: D1, bias arising from randomization processes; D2, bias due to deviations from intended intervention; D3, bias due to missing outcome data; D4, bias in measurement of outcome; D5, bias in selection of reported result; green circle represents low risk of bias, yellow circle indicates some concerns of risk of bias, red circle reports high risk of bias

Using the Cochrane risk of bias assessment (Fig 2), 10 articles [12, 13, 26–30, 33] used computer-generated random number tables; 3 article [26] used allocation concealment; 2 article [26] was double-blind, and all articles reported loss to follow-up during the study process, indicating relatively high methodological quality; 2 articles [15, 27] were rated A, and the remaining articles[12, 13, 27–30, 33, 49] were rated B (Table 2).

**Table 2:** Risk of Bias Assessment of Included Studies

First author	Random Sequence Generation	Assignment hiding	Masking (in scientific experiments)		Description of lost visits	Results data completeness	Selective Reporting of results	Others Bias	Quality level (grade)
			Subjects and implementers of the study	Evaluator					
Giuseppe Pichierri[9, 30]	Low	Unclear	Unclear	High	Low	Low	Low	Low	B
Jahouh et al 2021 [15, 16]	Low	Low	Low	Low	Low	Low	Low	Low	A
Katsunari Sato[3, 21]	High	Unclear	High	Low	Low	Low	Low	Low	B
Mosayeb Mozafari, PhD[4, 26]	Low	Low	Low	Low	Low	Low	Low	Low	A
Nadia Rizki Rahmawati[5, 27]	Low	Low	Unclear	Low	Low	Low	Low	Low	B
Pauline Maillot[6, 28]	Low	Unclear	Unclear	Unclear	Low	Low	Low	Low	B
Lim et al 2023 [3, 7, 29, 41]	Low	Low	High	High	Low	Low	Low	Low	B
Schoene,Daniel [12, 33]	Low	Unclear	High	Unclear	Low	Low	Low	Low	B
Suwicha Jirayucharoensa [8, 17]	Unclear	High	High	High	Low	Low	Low	Low	B
Zheng Jiaying [12, 13]	Low	Unclear	Unclear	Unclear	Low	Low	Low	Low	B
Nathalie Swinne [13, 14]	Low	Unclear	High	High	Low	Low	Low	Low	B
Maha Jahouh [56]	Low	Unclear	Unclear	Unclear	Low	Low	Low	Low	B
Hafsah Arshad [15, 49]	High	High	High	Unclear	Low	Low	Low	Low	B

Low = Low risk of bias; High = High risk of bias; Unclear = Unclear risk of bias

### Meta-analysis

According to different types of exergame interventions, the exergames were sub grouped into dance-based and action-based for subgroup analysis [57]. According to different intervention durations, the intervention periods were sub-grouped into 8 weeks and 8 weeks or more for subgroup analysis [58, 59]. This meta-analysis included 13 controlled studies in total and aimed to clarify and compare the effects of different types of exergames and different intervention durations on cognitive function in older adults with cognitive impairment. The standardized mean differences (SMD) and their confidence intervals for each study.

#### Cognitive function analysis

**Fig. 3:**
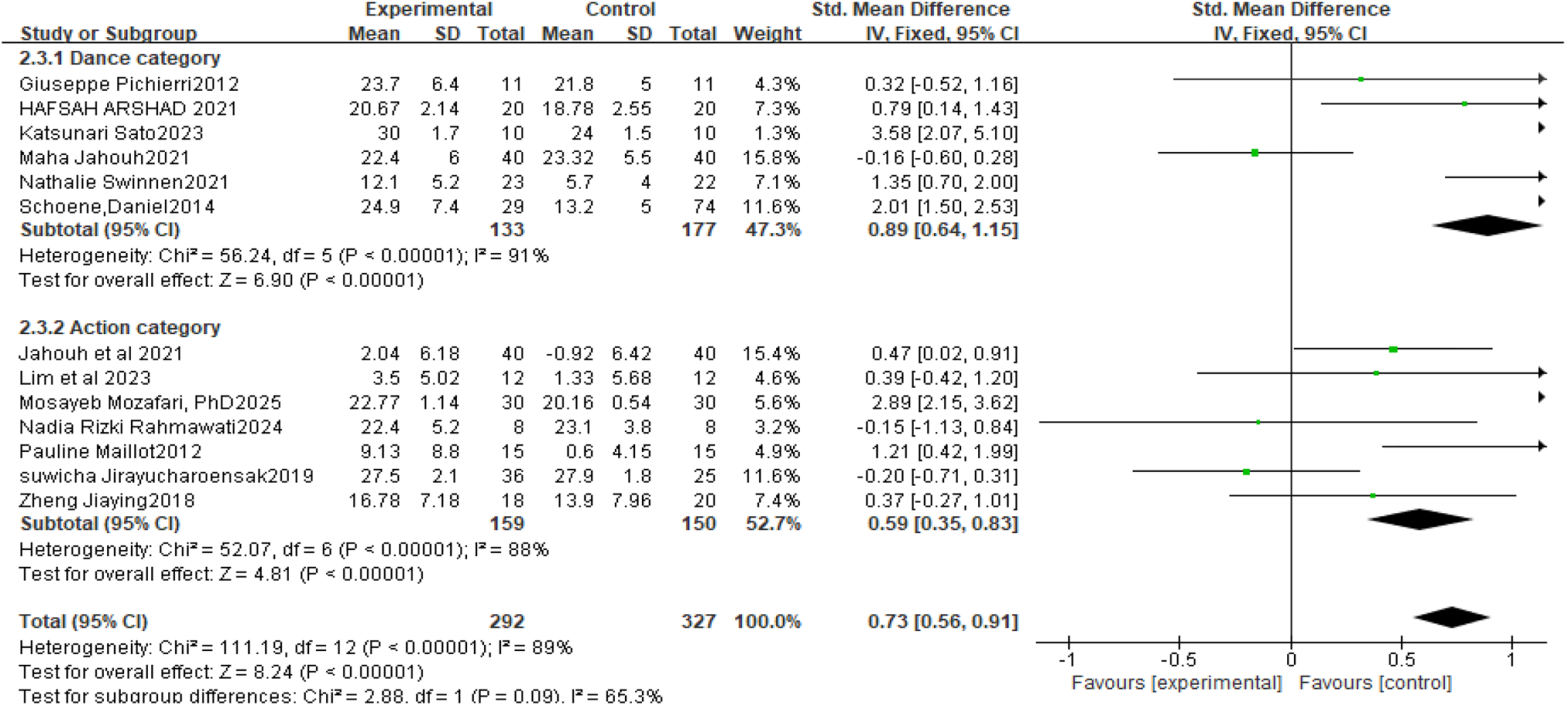
Cognitive Abilities of Elderly Patients with Cognitive Impairment Based on Dance and Motion-Sensing Game Interventions

Thirteen controlled studies were included. Statistical heterogeneity among the studies was high (P<0.001, I^2^=89%), so a random-effects model was used for data processing. Because the outcome measures across these thirteen studies were not completely consistent, standardized mean differences were used. The results showed that dance-type interventions were superior to movement-type [SMD=0.73, 95% CI (0.56, 0.91), P<0.0001; see Fig 3], and the difference was statistically significant. For movement-type, seven studies were included [SMD=0.59, 95% CI (0.35, 0.83), P<0.0001; see Fig 3], and the difference was not statistically significant, indicating that movement-type exergames have a consistent statistically significant effect on cognitive function in older adults. For dance-type, six studies were included; the treatment groups showed greater cognitive improvement than the control groups, and the difference was statistically significant [SMD=0.89, 95% CI (0.64, 1.15), P<0.0001; see Fig 3].

#### Intervention duration analysis

**Fig. 4:**
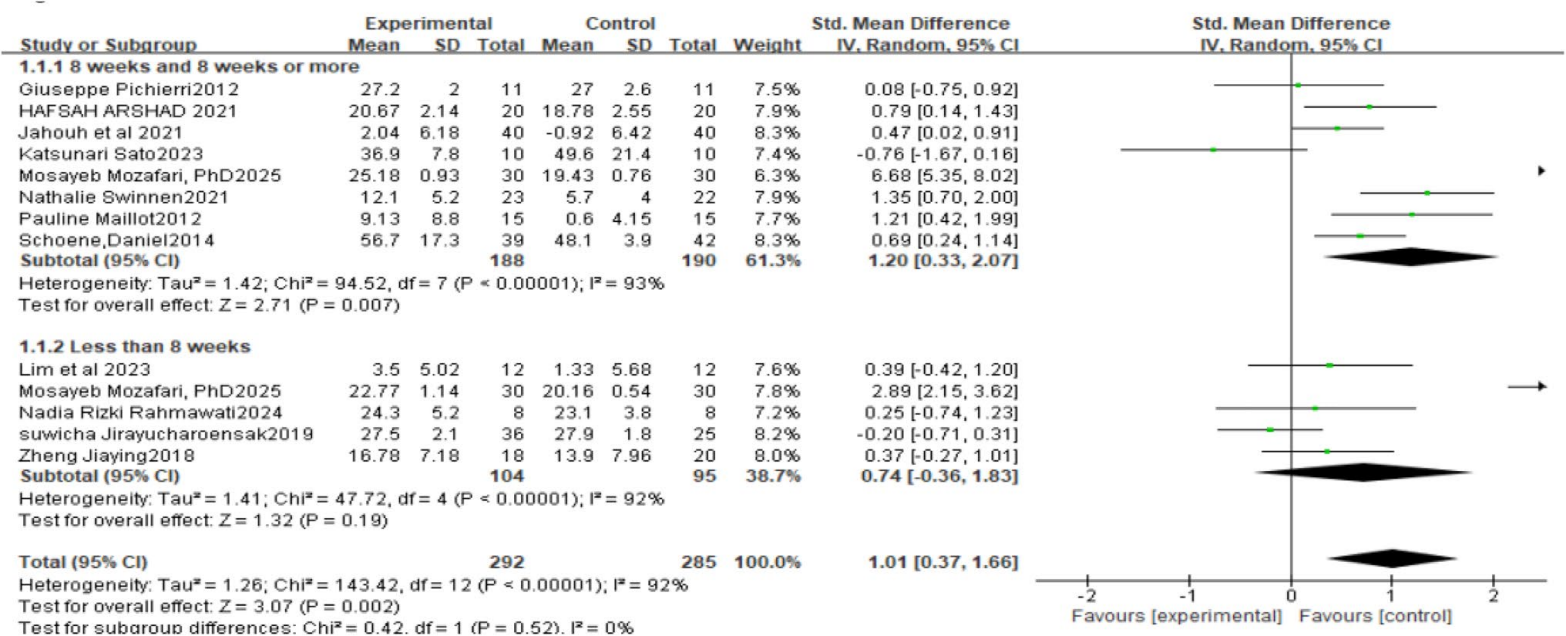
Effects on elderly people with cognitive impairment 8 weeks and 8weeks or more and less than 8 weeks of motion-sensing game intervention

A total of 13 controlled studies were included. Statistical heterogeneity among studies was high (P<0.001, I^2^=92%), so a random-effects model was used for the meta-analysis. The results showed that interventions of 8 weeks and longer were superior to those shorter than 8 weeks [SMD=1.01, 95% CI (0.37, 1.66), P=0.02; see Fig 4], and the difference was statistically significant. For durations of 8 weeks and longer, 8 studies were included [SMD=1.20, 95% CI (0.33, 2.07), P<0.01; see Fig 4], and the difference was statistically significant. For durations shorter than 8 weeks, 5 studies were included [SMD=0.74, 95% CI (-0.36, 1.83), P<0.001; see Fig 4], and the difference was not statistically significant.

#### Subgroup analysis tests

**Fig. 5:**
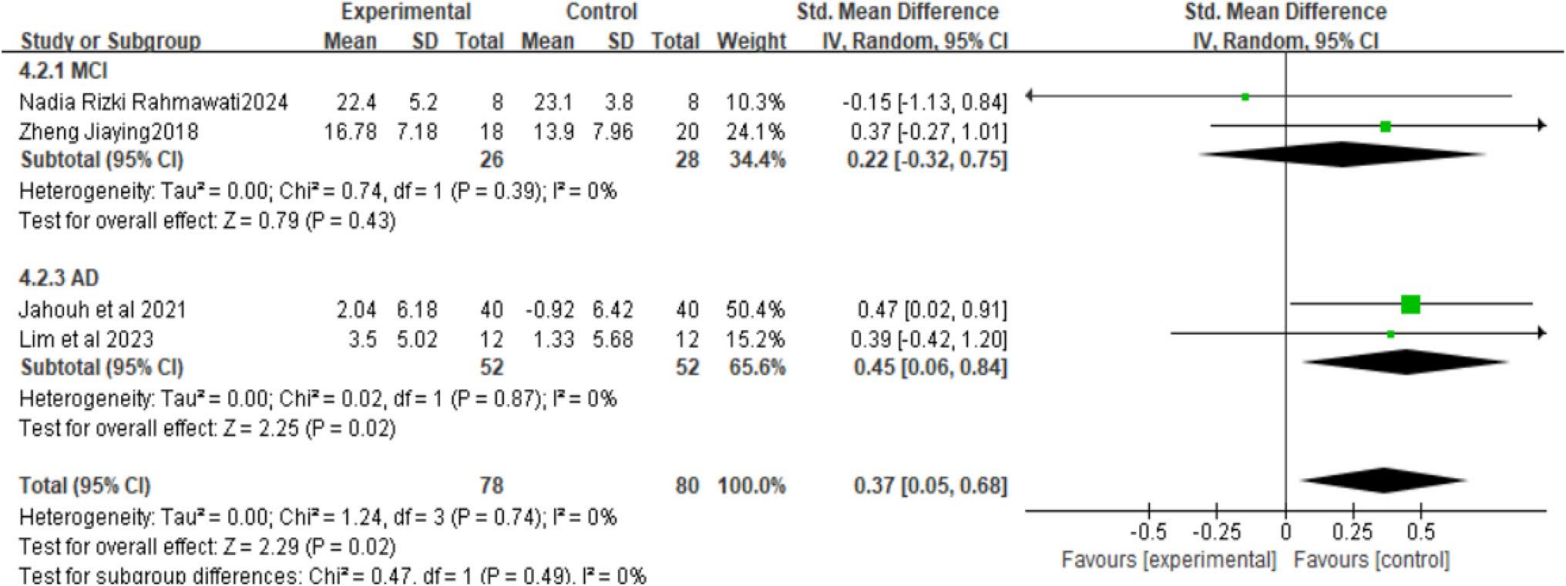
Meta-analysis of the cognitive effects of action-based motion-sensing games on elderly people with varying degrees of cognitive impairment

**Fig. 6:**
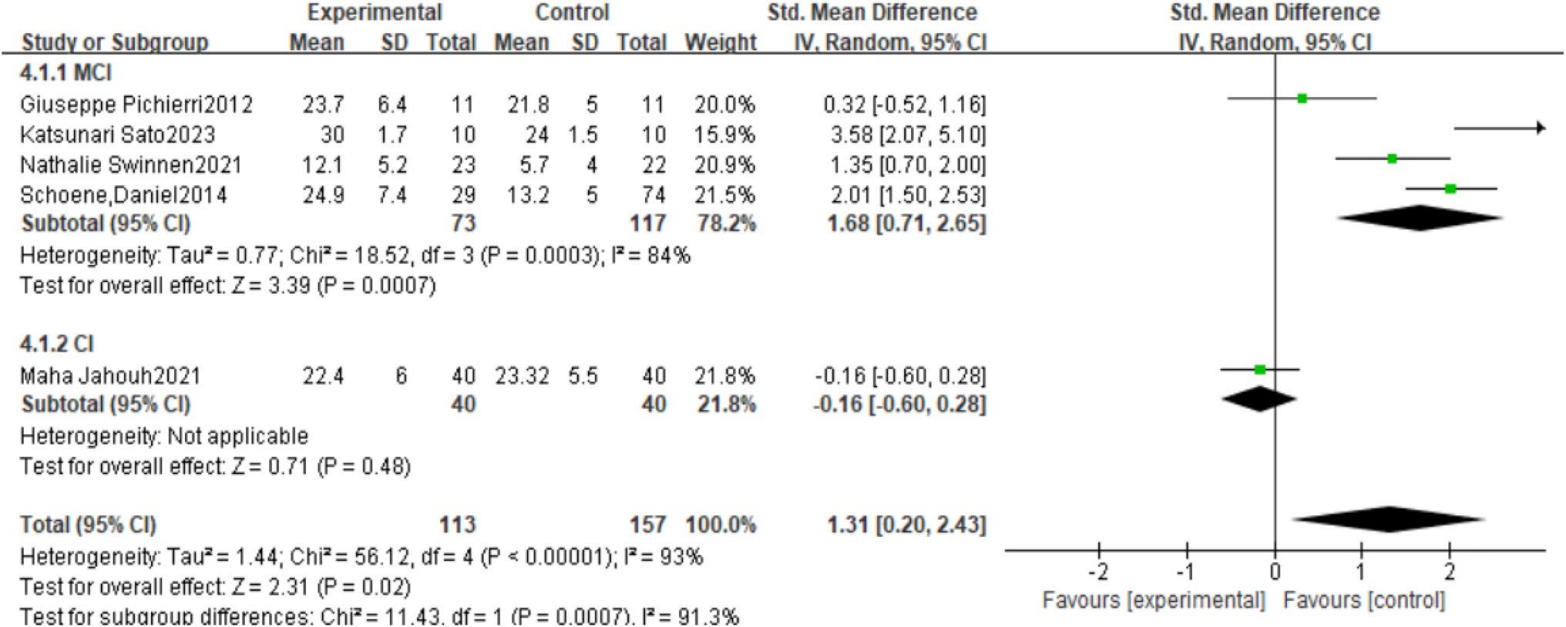
Meta-analysis of the cognitive effects of dance-based motion-sensing games on older adults with varying degrees of cognitive impairment

Studies found that motion-sensing games can effectively improve cognitive function in older adults. Because the forest plot showed high heterogeneity, and given the substantial heterogeneity, we further explored its sources. By cognitive impairment severity, AD represents the Alzheimer’s population, MCI represents the mild cognitive impairment population, and CI represents the cognitively impaired population. For intervention types, subgroup analysis results showed lower heterogeneity for action-type motion-sensing games (I^2^=0%), indicating consistency within this subgroup. The overall effect estimate for this subgroup was [SMD=0.37, 95%CI (0.05, 0.68), P<0.01, see Fig. 5], showing no significant difference between the two studies, and P<0.05, the difference was statistically significant. Dance-type interventions had higher heterogeneity (I^2^=93%, see Fig. 6). At the same time, regarding intervention duration, there remained high heterogeneity when grouping by cognitively impaired populations. But P<0.05, indicating statistical significance. High heterogeneity suggests that results may be influenced by differences in sample characteristics, publication time, study location, study design, or methodology.

Overall, action-type motion-sensing games produced better effects in intervening with the Alzheimer’s population than with the MCI population, while dance-type motion-sensing games were more effective for older adults with MCI than for the cognitively impaired population. Regarding intervention duration, regardless of length, interventions for the Alzheimer’s population were more effective than for the MCI population. Meanwhile, given the currently limited number of related studies and small sample sizes, further large-scale, multicenter studies are recommended to better reveal the true effects of the interventions and reduce the impact of heterogeneity.

#### Sensitivity analysis

A sensitivity analysis was conducted on the 13 included studies by altering study quality differences, inclusion criteria, statistical models, and choice of effect size, and re-running the meta-analysis. The pooled results did not change significantly, indicating that the meta-analysis findings of this study are relatively reliable.

### Publication bias assessment

**Fig. 7:**
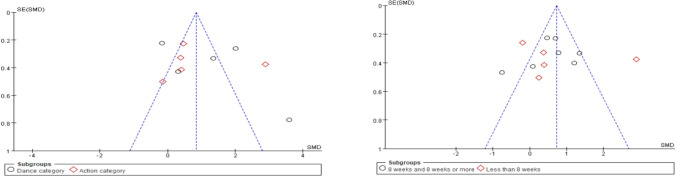
Sensitivity analysis forest plot

To assess potential publication bias, we constructed funnel plots (Fig. 7). In the absence of publication bias, study points should be symmetrically distributed around the pooled effect size and lie within the 95% confidence intervals. However, visual inspection of both funnel plots revealed clear asymmetry, with most studies clustered on the left side of the plots. This pattern suggests a possible lack of studies reporting smaller or negative effect sizes.

## 5GRADE results

Import the results of the meta-analysis into the GRADE pro software to assess the quality of evidence. The results show that TMT is high-quality evidence; MoCA is rated as moderate-quality evidence because allocation concealment was not used and it was not a randomized controlled trial; MMSE is assessed as low-quality evidence because it was not a randomized controlled trial, allocation concealment was not used, and blinding of intervention implementation was not performed (Table 3).

**Table 3:** Level of evidence of Meta-analysis results based on GRADE pro system

Outcome	Included studies(n)	Patients	Quality of evidence	Reasons
MMSE	8	343	○○○○ (Low)	Not an RCT, Unused allocated hidden, Did not blind the intervention implementation
MoCA	10	488	●●○○ (moderate)	Not an RCT, Unused allocated hidden
TMT	3	123	●●●○ (High)	Small sample size

## Discussion

### Intervention effects of exergames on older adults with cognitive impairment

#### Effects of different intervention types of exergames on cognition in older adults with cognitive impairment

This study used a meta-analysis to compare the effects of dance-based and action-based exergames on cognitive function in older adults with cognitive impairment. The results showed that dance-based exergames were significantly superior to action-based exergames in improving cognitive function. Specifically, regarding intervention type, the dance group’s MoCA scores were significantly higher than those of the action group (SMD = 1.04, 95% CI (0.36, 1.72), P = 0.03). The findings suggest that dance-based exergames may be more effective at enhancing cognitive function in older adults with cognitive impairment. At the same time, subgroup analysis showed that when further dividing the cognitively impaired population, dance-based interventions were more effective for those with mild cognitive impairment than for those with cognitive impairment [SMD = 1.31, 95% CI (0.20, 2.43), P < 0.05], while action-based interventions were more effective for people with Alzheimer’s disease than dance-based interventions [SMD = 0.37, 95% CI (0.05, 0.68), P < 0.01].

#### Effect of intervention duration on cognitive outcomes

The length of the intervention period also had a significant impact on the effects of exergames on different cognitive domains. The results showed that interventions lasting 8 weeks or longer were significantly superior to interventions of less than 8 weeks [SMD = 1.01, 95% CI (0.37, 1.66), P = 0.02]. At the same time, subgroup analysis revealed that when further dividing the cognitively impaired population, intervention durations both under 8 weeks and 8 weeks or longer produced better effects for people with Alzheimer’s disease than for those with mild cognitive impairment.

Although heterogeneity remained considerable after subgroup analyses due to differences in sample characteristics, publication time, study regions, study design, or methodology, the included studies were assessed using the GRADE approach; despite the high heterogeneity, our conclusions still have relatively high credibility.

### Mechanisms by which different types and durations of exergames affect cognition in older adults with cognitive impairment

Dance-based and action-based motion-sensing games show domain-specific differences in cognitive improvement, primarily due to their differing task demands and neural mechanisms [3, 60, 61]. Neuroimaging evidence indicates that brain changes induced by dance games mainly involve the basal ganglia–thalamocortical circuits (such as the putamen and globus pallidus), regions associated with procedural learning and rhythm processing [62]; in contrast, action games more frequently activate the dorsal attention network and the sensorimotor cortex, optimizing visuospatial processing and response inhibition [61]. Regarding long-term effects, dance games may provide more sustained cognitive protection. Sato’s pilot study found that MoCA scores in MCI patients increased after dance game training alongside enhanced prefrontal activity, suggesting a potential enhancement of neural reserve [48]. The benefits of action games, however, may depend more on continued training; Zhang’s study showed that some cognitive gains can diminish once training stops, indicating that the effects of action games are more reversible [63]. These differences suggest that dance games are better suited for interventions targeting global cognitive decline, while action games are more effective for strengthening specific cognitive domains (such as attention deficits) [48, 60]. Dance games primarily improve executive function, memory, and autonomic regulation through multitasking, rhythm synchronization, and musical stimulation, with mechanisms involving activation of basal ganglia–cortical circuits and HRV optimization [3, 62, 64]. Action games focus on rapid decision-making, sensorimotor integration, and challenge difficulty, enhancing processing speed, attention, and motor control, with mechanisms related to increased neural efficiency in attention networks and the sensorimotor cortex [60, 61]. Therefore, dance and action games produce different effects when intervening in older adults with cognitive impairment.

Regarding intervention duration, short-term interventions under 8 weeks mainly engage rapid neuroplasticity mechanisms, such as increased BDNF expression [65], whereas long-term interventions (8 weeks and longer) may induce more stable synaptic reorganization and neural network remodeling [66]. Animal studies show that 3xTg-AD mice exhibit reduced expression of synaptic markers at 12 weeks of age, followed by a compensatory increase [67], suggesting that different intervention durations may target different stages of neuroplasticity. Early intervention (e.g., starting at 10 weeks) versus late intervention (starting at 18 weeks) in 5xFAD mice showed different effects; an early 8-week composite pulsed rhythmic magnetic field intervention produced more significant cognitive improvement [68]. Therefore, the effects brought about by different intervention durations also vary [69].

### The mechanisms underlying cognitive impairment and Alzheimer’s disease are different

There are both continuous and stage-specific differences in the neural mechanisms of Alzheimer’s disease and mild cognitive impairment [69]. For example, studies using 18F-FDG PET to observe cerebral glucose metabolism have shown that AD patients exhibit significant metabolic reductions in multiple brain regions, especially the medial temporal and parietal areas, whereas MCI patients show metabolic declines that are relatively mild and localized. Xiong and colleagues further linked decreases in glucose metabolism with deterioration in specific cognitive domains, suggesting that changes in metabolic function are important biological markers of cognitive decline [69]. Recently, Zhang and others, based on large-scale resting-state fMRI data, proposed that AD patients and MCI converters show significant alterations in neural information storage times across different brain regions. AD patients and converters exhibited prolonged information storage times in lower-level regions, while stable patients with CI showed dysfunction in higher-level regions, leading to a weakened gradient of neural timescales [70]. These scale changes reflect disruption of excitation/inhibition balance and relate to the neurodynamic basis of cognitive decline, indicating differences in various neurophysiological processes during AD pathological progression. Therefore, the same type of intervention can have different effects on populations with different degrees of cognitive impairment [71].

### Limitations of the study

Although this study concluded that exergames have a positive intervention effect on older adults with cognitive impairment, several limitations remain: (1) Quality and quantity of included studies: the number and quality of studies included in this review are limited, and some studies may be at risk of bias. The presence of publication bias may lead to an overestimation of the pooled intervention effect. Because studies with null or negative results are less likely to be published, publication bias may result in an overestimated overall standardized mean difference (SMD). Therefore, the reported positive effects of different types of exergames on cognitive function in older adults should be interpreted with caution. To reduce publication bias in future research, prospective trial registration must be implemented and all results should be fully reported—regardless of statistical significance. In addition, some studies did not use blinded designs, which may affect the objectivity of the results. (2) None of the included articles addressed dose–response effects of exercise. Exercise intensity was controlled by participants’ subjective judgments, which limits our comprehensive assessment of the overall effect of the exercise interventions and makes it difficult to determine what exercise dose achieves optimal health benefits. Future studies could supplement with investigations of dose–response relationships to provide more targeted guidance for practical application. (3) Because the number of included studies and sample sizes in this review are small and heterogeneity is high, potential causes include differences in sample characteristics, publication dates, study regions, research designs, or methodologies. Other causes may be the use of different assessment scales for outcomes and varying degrees of cognitive impairment among included participants. Future research should include more similar studies, increase sample sizes, and strive to standardize study designs, population characteristics, and interventions to reduce heterogeneity and improve the reliability and generalizability of results. (4) Inconsistent outcome measures were observed; more comprehensive assessment scales and evaluation methods should be established. (5) This study searched literature in Chinese and English only, which may result in incomplete inclusion of other relevant publications.

## Conclusion

In summary, this study used meta-analysis to explore the cognitive effects of dance-type and movement-type exergames on older adults with cognitive impairment across different intervention durations, and concluded that exergames, as an emerging nonpharmacological intervention, can improve cognitive function in older adults with cognitive impairment to a certain extent. The effects were especially notable for dance-type exergames and longer intervention periods (12 weeks or more). This finding offers new ideas and methods for clinical intervention, and it is suggested that exergames may be considered as an adjunctive intervention in the rehabilitation of older adults with cognitive impairment. However, given the study’s limitations, clinical application should be cautious, taking into account patients’ specific circumstances and formulating individualized intervention plans.

## Supplementary Information

Below is the link to the electronic supplementary material.

Supplementary file1 (DOCX 1127 KB)

## Data Availability

No datasets were generated or analysed during the current study.
